# Lethal Synergism between Influenza and *Streptococcus pneumoniae*

**DOI:** 10.16966/2470-3176.114

**Published:** 2016-04-30

**Authors:** Jennifer M Rudd, Harshini K Ashar, Vincent TK Chow, Narasaraju Teluguakula

**Affiliations:** 1 Center for Veterinary Health Sciences, Oklahoma State University, OK, USA; 2 Department of Microbiology and Immunology, Yong Loo Lin School of Medicine, National University of Singapore, Singapore

**Keywords:** Bacterial co-infection, Influenza, *Streptococcus pneumoniae*, Pathogenesis, Therapeutics

## Abstract

The devastating synergism of bacterial pneumonia with influenza viral infections left its mark on the world over the last century. Although the details of pathogenesis remain unclear, the synergism is related to a variety of factors including pulmonary epithelial barrier damage which exposes receptors that influence bacterial adherence and the triggering of an exaggerated innate immune response and cytokine storm, which further acts to worsen the injury. Several therapeutics and combination therapies of antibiotics, anti-inflammatories including corticosteroids and toll-like receptor modifiers, and anti-virals are being discussed. This mini review summarizes recent developments in unearthing the pathogenesis of the lethal synergism of pneumococcal co-infection following influenza, as well as addresses potential therapeutic options and combinations of therapies currently being evaluated.

## Pandemic Co-infection: A History of Influenza Pandemics and Secondary Bacterial Pneumonia

Beginning in 1918, as World War I was coming to a close, influenza pandemic occurred resulting in an estimated 50 million deaths worldwide [1–4]. In just a few short years, the pandemic had killed well over double the number of people who had died due to World War I. Termed the “Spanish Flu”, this pandemic resulted in excessive mortality well beyond the expected seasonal influenza and targeted young, otherwise healthy adults with a swiftly deadly disease course [1,5]. Based on preserved lung tissue sections and autopsy analyses, 95% of these deaths were attributed to co-infections during the 1918 flu pandemic [5,6]. Since 1918, three more influenza pandemics have occurred, two with disproportionate rates of mortality. The H2N2 “Asian Flu” pandemic of 1957–1958 and the H3N2 “Hong Kong” Flu of 1968 [7]. In 1968, the Hong Kong Flu hit the world in two waves-the first causing excessive mortality in North America, and the second wave affecting Europe, Asia and Africa between 1968 and 1970 [8,9]. More recently, in 2009, the triple reassortment H1N1 virus, termed the “Swine Flu”, had killed roughly 285,400 people worldwide by its completion in 2010 [2,5]. Throughout all these pandemics, co-infections continued to play key role in lethality, making it crucial to consider these bacterial co-pathogens when planning for a pandemic [10,11].

In an extensive review of influenza and bacterial co-infections from the 20^th^ century, several more common pathogens were identified including *Streptococcus pneumoniae, Haemophilus influenzae, Staphylococcus spp*. (in particular *S. aureus*), and other *Streptococcus spp.* [12]. Beyond the threat of high rates co-infections in pandemics, bacterial-super infections also contribute to about 65,000 deaths by seasonal influenza virus infections every year in the United States [2,12], although the rates of bacterial co-infections were found to be considerably higher during a pandemic than during the seasonal influenza period-of those bacterial co-infections, 41% were identified as *S. pneumoniae*, followed by 25% *Staphylococcus spp.*, 16% other *Streptococcus spp.*, and about 13% *H. influenza* [12]. Despite *S. pneumoniae* emerging as the predominant strain in 1918, during the 1957 pandemic, the clinical presentation of the disease shifted to a fulminant pneumonia with severe pulmonary edema and hemorrhage resulting in rapid death. This was soon attributed to principal co-infection with *S. aureus* [13]. By the following pandemic in the late 1960’s, *S. pneumoniae* had again emerged as the predominant bacterial co-pathogen.

*S. pneumoniae*, also termed pneumococcus, is a gram-positive diplococci that commonly colonize the upper respiratory tract of 20–50% of healthy children and 8–30% of healthy adults [14]. Although generally asymptomatic when colonizing the nasopharynx, pneumococcus is also the most frequently seen bacterial agent in bacterial meningitis, otitis media, sepsis and all community-acquired pneumonia [14] and is correlated with an increase in intensive care unit hospitalizations and death [2]. Pneumococcal disease is difficult to classify because of the diverse nature of its various strains and serotypes which affect disease outcomes, co-infection models and transmission [15]. Pneumococci can express one of over 90 capsule types which greatly alter their pathogenicity, and makes development of effective vaccines and therapies difficult [15–17]. Diagnosis is also quite difficult, as many of the bacterial pathogens seen in co-infection, *S. pneumoniae* in particular, regularly colonizes the nasopharynx [1]. As the predominant co-pathogen in influenza co-infection, this mini review will focus on the proposed contributors to the pathogenesis of the synergistic co-infection of *S. pneumoniae* with influenza, as well as several therapeutic options being considered at this time.

## The Complexity of Co-infection: Why are Influenza Viruses and *Streptococcus pneumoniae* Lethally Synergistic?

### Pulmonary epithelial barrier damage

It has been shown that mice exposed to influenza have hyper inflammatory responses with increased bacterial burdens and decreased pulmonary clearance of *S. pneumoniae* following co-infection compared to controls [18]. Although the exact mechanisms behind the lethal synergism seen with co-infection remain unclear, numerous causative pathways and pathology have been researched to establish the connection. Influenza infection damages the host by causing alveolar epithelial damage, surfactant disruption and resultant obstruction of small airways by sloughed cells, mucus and other debris [14,19]. The damage to the respiratory epithelium leads to exposure of the underlying basement membrane and progenitor epithelial cells, resulting in an inability of the respiratory epithelium to repair itself and re-proliferate [20]. As epithelial damage is worsened, a rise in lethality, likely due to bacteremia, is appreciated [20,21]. Exposure of the basement membrane and fibrin also increase bacterial adherence [4]. Pandemic viral infections inflict high cytotoxicity on the alveolar epithelium, which could possibly contribute to the increase in proportions of co-infections seen at these times [2,20]. In addition, influenza infection also causes a decrease in mucociliary clearance and coordination, resulting in failure of removal of bacteria prior to the adherence to the damaged surfaces in the lung [14].

### Receptor exposure and bacterial adherence

The desialylation by influenza viral neuraminidase also participates in bacterial adherence to epithelial cells. Sialylated mucins act as decoy receptors for the bacteria [1,3,4,22]. The effects that co-infection has on the recognition of microbial glycans by lectins enhances this pneumococcal adhesion, making patients with influenza more susceptible to secondary pneumonia [22]. Damage of epithelial cells also expose glycanson their surface, thus enhancing bacterial adherence [22]. A variety of proteins are altered and displayed on epithelial cells following influenza virus infections, such as platelet activating factor receptor (PAFr), that promote bacterial adherence and disease [1,23]. Pneumococci also have a variety of virulence factors that allow adherence to these newly exposed receptors on damaged epithelium, laminin and fibrin, including pneumococcal surface protein A (PsaP) and pneumococcal serine-rich repeat protein (PsrP) [16]. PsaP is a lipoprotein pneumococcal antigen that aids in adherence to nasopharyngeal epithelial cells via E-cadherin, while PsrP is a lung-specific adherin [24].

### The innate response: can you have too much of a good thing?

**Figure 1:**
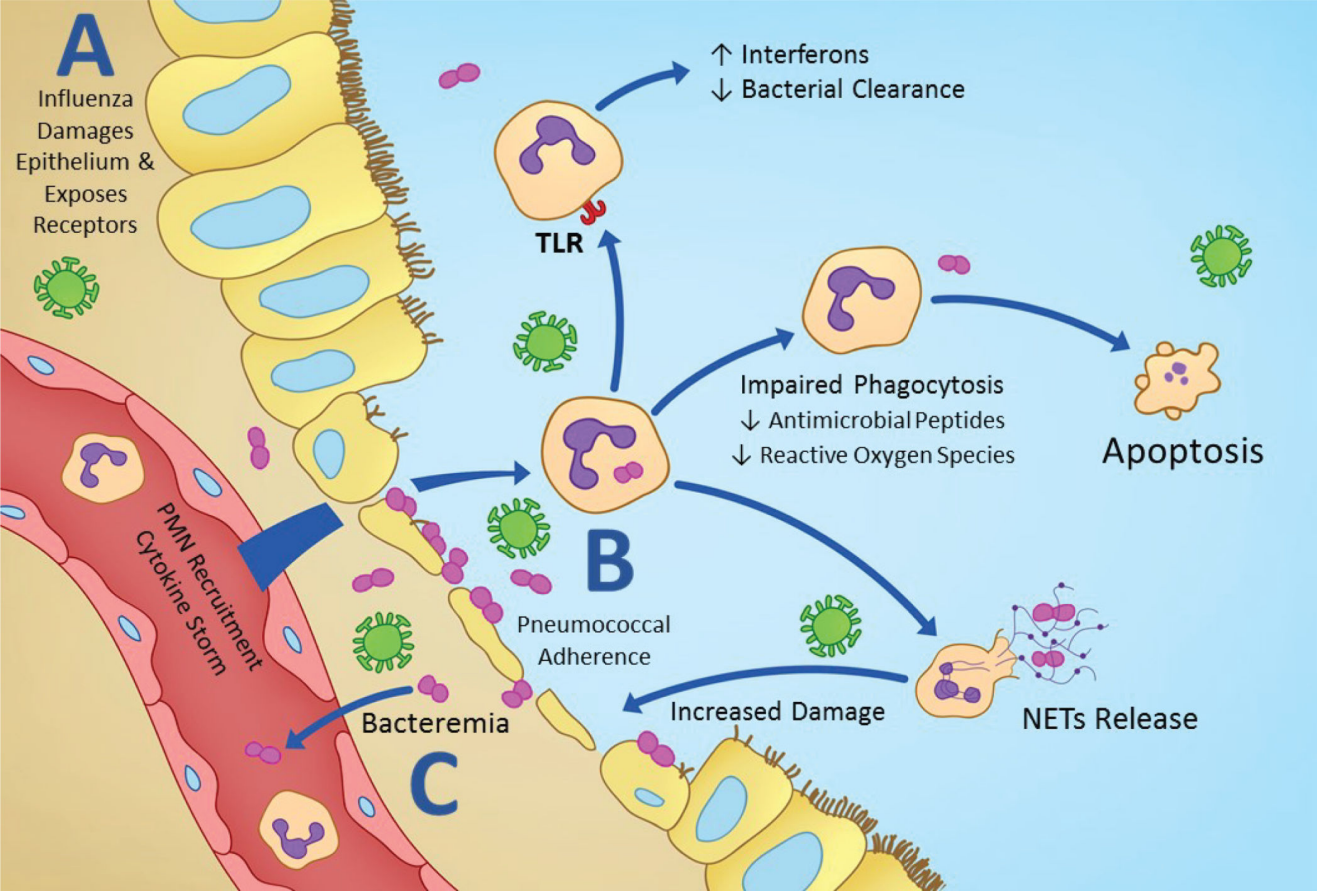
Neutrophils are key players in co-infection pathogenesis (A) Influenza damages airway epithelium and exposes receptors priming for bacterial adherence; *S. pneumoniae* adheres to damaged epithelium and is able to migrate through pulmonary epithelium. (B) Sentinel cells detect pathogens and damaged cells and recruit neutrophils through a chemotactic gradient for phagocytosis and bacterial killing; Neutrophils contribute to immunopathology through a variety of mechanisms as illustrated. (C) Worsened epithelial and endothelial damage due to coinfection results in bacteremia.

Several studies have highlighted exaggerated immune responses in contributing to the synergism during bacterial co-infection. Among innate immune cells, high neutrophil influx has been linked with increased immunopathology in bacterial super infections following influenza (Figure 1) [25]. Neutrophils are short lived and terminally differentiated cells, primarily involved in phagocytic clearance of the bacteria. The ingested bacteria are destroyed through the generation of potent oxidants after activation of the NADPH oxidase complex (respiratory burst) or by lytic enzymes and antimicrobial peptides within the phagolysosome. After bacterial co-infection, neutrophil numbers become excessive within hours, but macrophages and dendritic cells do not share the same disproportionate increase [26]. Myeloperoxidase measurements do not increase at the same rate as the neutrophil quantity, suggesting that these rapidly recruited neutrophils will not have the same antibacterial function that the initial responders did [26]. Functional impairment of neutrophils is seen through several capacities. Phagocytosis has been shown to be decreased in both neutrophils and macrophages following influenza infection [25,26] and several pathways to this reduction have been evaluated including resistance to phagocytic granule components [27], and the down regulation of the MARCO receptor due to interferon production [4,28,29]. Neutrophils and macrophages also have a marked decrease in reactive oxygen species following co-infection [29]. These cells can kill pathogens through oxidative burst, which creates toxic reactive oxygen species through NADPH oxidase complex or myeloperoxidase. Gram positive bacteria such as *S. pneumoniae* can have a bacterial superoxide dismutase that can protect the pathogen from these toxic species [27].

Neutrophils can potentially cause worsened inflammatory disease through the release of neutrophil extracellular traps (NETs). We have previously shown that excessive neutrophils and NETs contribute to alveolar-capillary damage after influenza challenge in mice. NETs formation is dependent on redox enzyme activities [30]. NETs were first identified as a process of cell death that released DNA, histones and granular proteins such as elastase and myeloperoxidase to entrap and kill pathogens [31]. Since the initial identification of NETs, they have also been shown to be detrimental to the host-particularly through histones which induce endothelial and epithelial cell damage and worsened disease [32]. Further, using pneumococcal super infection following influenza, an extensive accumulation of NETs was recognized, especially in the damaged areas of the lungs, indicating their potential role in tissue injury. Moreover, NETs released during pneumococcal super infection did not show any bactericidal or fungicidal activities [33,34]. Our recent studies have shown that NETs generation is dependent on the pneumococcal capsule thickness and varies with the different serotype infections. The increase in thickness of the capsule results in enhanced tissue damage and lung pathology [17]. NETs have been identified in various inflammatory disease models other than pneumococcal pulmonary co-infection such as co-infection of otitis media and sepsis [35,36]. Although the complete pathway for NETs induction has yet to be discovered, *S. pneumoniae* has been shown to induce NETs through an enzyme called α-enolasae [37]. Paradoxically, a pneumococcal endonuclease, EndA, has been identified as an important virulence factor through its ability to degrade NETs and diminish their bactericidal response [38]. As with many other areas of the complex pathogenesis of co-infection, it appears that NETs too must be balanced between positive effects and those that are detrimental to the host.

Apoptosis of various cell types also appears to be affected by bacterial co-infection after influenza. Monocytes express a TNF-related apoptosis-inducing ligand (TRAIL) that can be blocked through CCR2 blockage and result in decreased bacterial load and protection if administered prior to co-infection [39]. *In vitro*, influenza virus has been shown to accelerate neutrophil apoptosis by enhancing Fas expression and activating caspase, decreasing neutrophil survival [40]. The significant neutrophil influx triggered by various viral and bacterial toxins such as PB1-F2 in a co-infection result in a cytokine storm and can lead to a severely damaging hyper inflammatory response which can be seen histopathologically as excessive neutrophilia, sloughing epithelium, hemorrhage, obstructed airways, pleuritic and large areas of lung consolidation [26].

### Toll-like receptors and their contribution to immunopathology and interferon signaling

Toll-like receptors are an important part of the innate immune response and recognize conserved patterns in a variety of pathogens. Upon recognition, these receptors trigger a series of events resulting in activation of the innate immune response through production of various pro-inflammatory chemokines, cytokines, interferons and recruitment of those innate responders such as the neutrophils and macrophages [41]. In particular, these TLRs can recognize cellular wall components of gram-positive organisms, such as those in *S. pneumoniae* [42]. Influenza induces expression of toll-like receptors, such as TLR3 which acts to recognize RNA and DNA of pathogens after phagocytosis, and this not only sensitizes cells to secondary infection with pneumococcal pneumonia, but also decreases bacterial clearance and increases type I interferons, which have been shown to negatively affect survival in a murine model [43,44]. In addition to impairment of phagocytosis, production of interferons after recognition of pathogens by TLRs plays a large role in pathogenesis of co-infection as well. Type I and II interferons are produced following recognition of viral nucleic acids by toll-like receptors (TLRs) [1]. The induction of type I interferon during a primary nonlethal influenza infection was shown to be sufficient to promote lethality with co-infection of *S. pneumoniae* [45]. In addition, mice deficient in type I interferon receptor signaling has improved survival and bacterial clearance [46]. One mechanism by which type I interferon release in response to influenza infection results in worsened bacterial super infection is through the suppression of γδ T cell production of interleukin-17 (IL-17) [45]. γδ T cells in the lung act as specialized innate responders and normally produce the majority of IL-17 in response to a variety of viral and bacterial infections [45,47,48] which can suppress the effects of bacterial super infection. If type I interferon signaling is up regulated and IL-17 production suppressed through decreased γδ T cell function, bacterial colonization in the lungs is increased causing in deteriorated pathology and disease [45]. With interferon signaling increase, an impaired production of the neutrophil attractants CXCL1 and CXCL2 was noted following co-infection. This may explain some of the impaired neutrophil response to the early phase of co-infection [46]. Pneumolysin, a cytolytic toxin of *S. pneumoniae*, induces substantial inflammation through activation of TLR4 [49]. TLR2 is also an important mediator of the damage associated with pneumococcal pneumonia [50]. As discussed, the innate immune response is necessary early in the disease course, but can result in worsened pathology if the response remains elevated for too long. Identifying the pathways most involved in this synergism and filling in the gaps with the pathology of the disease will not only improve our general knowledge in all co-infections, but, more importantly help identify therapeutic targets to improve clinical outcome in those affected.

## Current Prospective Therapeutics and the Efficacy of Combination Therapies

### Antibiotics and combination therapies

Due to the complex nature of co-infection, a wide variety of therapeutic options and combinations of therapy are being evaluated for efficacy in a dual infection model of influenza A virus with subsequent pneumococcal infection. Combination therapies suggest the best results at this time, with one element of the combination being antibiotic therapy. Several classes of antibiotics have been evaluated. Although β-lactams were initially considered a mainstay of treatment for pneumococcal pneumonia, it has been shown well over the last decade that standalone therapies are no longer ideal and that combinations with macrolides and fluoroquinolones are more effective, especially in light of emerging antibiotic resistance [51–53]. Macrolides such as azithromycin and clarithromycin are bacteriostatic and work by binding the 50S ribosomal subunit, thereby inhibiting protein synthesis. In addition to their antimicrobial effects, macrolides also have an immunomodulatory effect, which poses an additional benefit in combatting superinfections. Azithromycin in particular has been shown to improve survival in a mouse model of influenza and pneumococcal dual infection with almost double the survival rate than ampicillin (92% versus 56%) as well as improved outcomes over clindamycin [54]. Combination ampicillin and azithromycin for treatment of pneumococcal pneumonia not only decreases lung inflammation, but also decreases pulmonary vascular permeability and increases bacterial clearance, limiting the chances of septicemia [55]. A lower number of inflammatory cells and proinflammatory cytokines are seen with macrolide treatment than standalone β-lactams as well as less severe lung histopathology-as this antibiotic is bacteriostatic, the reduction in an otherwise exacerbated inflammatory response seen with β-lactam therapy may be due to lessening in bacterial lysis [50,54]. Another study comparing the effects of moxifloxacin, a bactericidal drug, with azithromycin in a murine model of acute bacterial rhinosinusitis supports this as the azithromycin treatment resulted in rapid bacterial clearance and reduced inflammation compared with the relatively limited effect of moxifloxacin [56]. Further evaluation of the potential negative effects of azithromycin in human disease is still needed, but a 2015 study evaluating cardiotoxicity of azithromycin in community-acquired pneumonia (CAP) showed that the QT prolongation suggested to be an adverse effect of therapy was not associated with treatment, but instead with the disease of pneumonia, regardless of the therapy administered [57].

### Anti-inflammatories

The use of corticosteroids in treatment of bacterial infections is always a hot topic and one heavily debated. On the one hand, some argue that the use of an immune inhibitor in combination with an antibiotic to reduce the bacterial burden can more effectively control the exaggerated inflammatory response seen in co-infection and that the use of steroids should improve survival rates. In a murine model, this seems to hold true-a susceptible murine model for the 2009 H1N1 pandemic showed that dexamethasone significantly improved survival rate and acute lung injury [58]. A reduction in the proinflammatory cytokine storm, and improved clinical outcomes was associated with combination treatment of dexamethasone and azithromycin in mice [26]. However, what is most concerning with corticosteroids was highlighted in a retrospective cohort study from 2011 in which the early use of glucocorticoids was significantly linked with the development of more severe disease versus patients who did not receive the drug in pandemic H1N1 [59]. The *in vivo* benefits in human disease, particularly in a pandemic setting, are clearly still up for debate.

Toll-like receptor agonists and antagonists are a relatively new area showing promise as a potential combination therapeutic for pneumococcal co-infection. Special attention has been given to TLR2, which has been shown to mediate the extensive tissue damage, lung necrosis and mortality seen after bactericidal treatment of pneumococcal pneumonia in a murine co-infection model [50]. This mediation was independent of TLR4 or the pneumococcal virulence factor, pneumolysin. TLR2 also plays a role in transmission of disease, likely with a multitude of other factors-when a TLR2 agonist (Pam3Cys) was administered in a murine model of co-infection, contact transmission was diminished as well as inflammation and bacterial shedding [41]. A TLR2 agonist was again seen to reduce the severity of pneumococcal infection post-influenza in a murine model by decreasing bacterial loads and pro-inflammatory cytokines, subsequently leading to decreased vascular permeability and reduced bacteremia [60]. Macrophage-activating lipopeptide 2 (MALP-2) is a TLR2/6 agonist that, when administered prior to pneumococcal co-infection, increases proinflammatory cytokine and chemokine release and enhances neutrophil recruitment without creating excessive inflammation, so also reduces bacterial loads and improves survival [61]. Like TLR2 agonists, TLR5, or flagellin, agonists also act as immunostimulants. Given in combination with an antibiotic, flagellin will decrease bacterial load and boost antibiotic activity by stimulating CXCL1 to recruit neutrophils and reduce bacteremia [62]. TLR3 also participates in the immunostimulatory response when stimulated by pneumococcal RNA. TLR3 acts through TRIF to secrete IL-12. In a co-infection, influenza virus up regulates TLR3 in dendritic cells, which helps prime the cells for recognition of pneumococcal disease [43]. In another study, a TLR4 agonist, UT12, showed promise in improving clinical outcome and disease in a murine coinfection model after hastening the macrophage recruitment response [63]. Modulating TLRs is an interesting approach to understanding the pathogenesis of co-infection and, with further evaluation, may provide some promising combination therapies to attempt. The timing of therapy and its clinical relevance should still be carefully considered, as this therapy is effective when administered after influenza infection, but prior to secondary infection.

The role of γδ T cells in interferon signaling and IL-17 production is also being explored as a therapeutic for bacterial super infections. Since super infected mice inhibit IL-17, resulting in worsened bacterial replication and disease, the administration of recombinant IL-17 in these mice has improved bacterial clearance indicating that induction of IL-17 remains a potential novel therapy [45]. In a recent study, recombinant IL-17F was administered just prior to *S. pneumoniae* infection in a murine model and the therapy resulted in decreased bacterial colonization in the lungs [64]. In general, modulation of IFN-I signaling, IL-17 production and the function of γδ T cells all remain intriguing areas of study for treatment of dual infections.

### Other potential therapeutics

Multiple other therapies are being evaluated as well. Anti-virals are a mainstay of treatment and many are looking for alternatives to oseltamivir. Peramivir is a neuraminidase inhibitor that reduced mortality in co-infected mice better than oseltamivir by inhibiting viral replication resulting in improved bacterial clearance and survival [65]. Although oseltamivir has shown effectiveness to both viral and bacterial neuraminidase, peramivir only seems to inhibit viral neuraminidase [65,66]. Another neuraminidase inhibiting compound, artocarpin, was shown to have a bactericidal effect *in vitro*, reducing pneumococcal viability by a factor of over 1000, and reduced biofilm formation [66]. Several agents to reduce vascular leakage have also been evaluated with varying effectiveness including Slit2N, vasculotide, atrial natriuretic peptide, S1P, activated protein C, and doxycycline [21,67]. Mathieu, et al. [68] has started evaluating the use of nanoparticles carrying a plant virus coat protein and ssRNA that trigger a strong innate immune response in the lung during a co-infection. Vaccinations are also a key area of research, especially when considering the effect these vaccinations may have in pandemic preparedness. Pneumococcal capsular polysaccharide conjugate vaccines have been shown to be very effective (100%) against otherwise lethal pneumococcal disease, but in co-infection, the results are not as promising with less than 40% survival with vaccination in a murine model [69]. The value of the current vaccine is evident already though, with the vaccine being 84–94% efficacious against the serotypes included and reducing the severity of disease and risk for hospitalization in those affected [4]. In the U.S. alone, we have seen a 39% reduction in clinical pneumonia in children since the vaccine has been introduced [70]. Imagine how effective the current vaccine will be once it’s more available in developing countries.

## Conclusions

Co-infection of *S. pneumoniae* with influenza promises to be a relevant disease for many years to come. Despite the many recent advances in our knowledge base regarding the disease, the complexity of pathogenesis implies that an effective “shotgun” approach to therapy is doubtful and a fine-tuned combination of antimicrobial agents with immunomodulators is likely to be more effective when treating the disease. Because of the expansive diversity in both influenza viral strains and pneumococcal disease and their ever-changing patterns of resistance and survival, therapy effective for one combination may not consistently work for all. This review touches on a few approaches to consider in therapeutic design, but continued discovery will be needed to better prepare for the next pandemic.
